# Prognostic factors in IUI

**DOI:** 10.5935/1518-0557.20180075

**Published:** 2019

**Authors:** Gulam Bahadur, Roy Homburg

**Affiliations:** 1 Reproductive Medicine Unit, North Middlesex University Hospital, Old Admin Block, Sterling Way, London N18 1QX, UK; 2 Homerton Fertility Unit, Homerton University Hospital, Homerton Row, London E9 6SR, UK

Dear Editor, 

Prognostic factors in IUI analyses is a subject both topical and of importance if pregnancy rates in IUI are to be improved. Although the recent retrospective study (Sicchieri *et al*., 2018) aims to decipher the various contributors to IUI pregnancy rates, such as patient age, cause of infertility, ovulation induction method, number of mature follicles and sperm with progressive motility, the overall pregnancy rate of 7.59%/cycle was disappointing and this may give a wrong perception of IUI practice. The UK IUI pregnancy rates averaged around 13.5%/cycle and some clinics exceeding 20%/cycle. It also means that with every 100 women seeking first line treatment as IUI, 25-35% of the cohort will become pregnant (Bahadur *et al*., 2016a). We even demonstrated how it was possible to overcome the severe oligozoospermic effect by utilising a consecutive ejaculate (Bahadur *et al*., 2016b). Crucial to IUI success was to switch over to gonadotropin stimulated cycles, aiming for 2-3 follicles, but with a strict cancellation policy to protect the patient from OHSS and multiple births. In more complex and older patients, combination stimulation regimes can be tried. The cost-effective data clearly shows that more pregnancies can occur with gonadotropin stimulated cycles (Peeraer *et al*., 2018), or where greater than 3 million motile progressive sperm are inseminated. Newer, well-constructed RCT's provide further strong support for using IUI as first treatment option based on high-quality evidence (Bensdorp *et al*., 2015; Farquhar *et al*., 2018; Nandi *et al*., 2017). Sicchieri *et al.* (2018) rightly draw attention to affordability issues in most parts of the world, and we now know that over 50% of women undergoing IVF do not need IVF (Malchau *et al*., 2017). The way forward is to achieve IUI pregnancies earlier, paying attention to numerous prognostic factors and tailoring the stimulation method for IUI. In particular, these are associated with the use of gonadotrophin stimulation, having greater than 3 million motile progressive sperm in the insemination, having 2-3 follicles, and above all ensuring safe practice against OHSS and multiple births with a strict cancellation policy in place. Secondly, clinics should monitor their outcomes in real time using a dedicated database. Our proposals should help steer the global use of IUI towards a more effective and efficient first line treatment option.
